# Portosystemic shunt surgery in the era of TIPS: imaging-based planning of the surgical approach

**DOI:** 10.1007/s00261-020-02599-z

**Published:** 2020-06-05

**Authors:** Uli Fehrenbach, Safak Gül-Klein, Miguel de Sousa Mendes, Ingo Steffen, Julienne Stern, Dominik Geisel, Gero Puhl, Timm Denecke

**Affiliations:** 1 Department of Radiology, Charité – Universitätsmedizin Berlin, Augustenburger Platz 1, Berlin, 13353 Germany; 2 Department of Surgery, Campus Charité Mitte | Campus Virchow-Klinikum, Charité – Universitätsmedizin Berlin, corporate member of Freie Universität Berlin, Humboldt-Universität zu Berlin, and Berlin Institute of Health, Berlin, Germany; 3 Department of Radiology, Krankenhaus Nordwest, Frankfurt am Main, Germany; 4 Department of Surgery, Asklepios Klnik Altona, Hamburg, Germany; 5 Department of Diagnostic and Interventional Radiology, Universitätsklinikum Leipzig, Leipzig, Germany

**Keywords:** Portosystemic shunt surgery, Magnetic resonance imaging, Computed tomography, Portal hypertension

## Abstract

**Purpose:**

With the spread of transjugular intrahepatic portosystemic shunts (TIPS), portosystemic shunt surgery (PSSS) has decreased and leaves more complex patients with great demands for accurate preoperative planning. The aim was to evaluate the role of imaging for predicting the most suitable PSSS approach.

**Material and methods:**

Forty-four patients who underwent PSSS (2002 to 2013) were examined by contrast-enhanced CT (*n* = 33) and/or MRI (*n* = 15) prior to surgery. Imaging was analyzed independently by two observers (O1 and O2) with different levels of experience (O1 > O2). They recommended two shunting techniques (vessels and anastomotic variant) for each patient and ranked them according to their appropriateness and complexity. Findings were compared with the actually performed shunt procedure and its outcome.

**Results:**

The first two choices taken together covered the performed PSSS regarding vessels in 88%/100% (CT/MRI, O1) and 76%/73% (O2); and vessels + anastomosis in 79%/73% (O1) and 67%/60% (O2). The prediction of complex surgical procedures (resection of interposing structures, additional thrombectomy, use of a collateral vessel, and use of a graft interposition) was confirmed in 87%, resulting in 80% sensitivity and 96% specificity. Larger shunt vessel distances were associated with therapy failure (*p* = 0.030) and a vessel distance of ≥ 20 mm was identified as optimal cutoff, in which a graft interposition was used. There was no significant difference between MRI and CT in predicting the intraoperative decisions (*p* = 0.294 to 1.000).

**Conclusion:**

Preoperative imaging and an experienced radiologist can guide surgeons in PSSS. CT and MRI provide the information necessary to identify technically feasible variants and complicating factors.

**Electronic supplementary material:**

The online version of this article (10.1007/s00261-020-02599-z) contains supplementary material, which is available to authorized users.

## Introduction

Transjugular intrahepatic portosystemic shunt (TIPS) has become the most common intervention used today to treat intrahepatic portal hypertension, PHT [1]. Before even being proved to be better than  portosystemic shunt surgery (PSSS), TIPS quickly spread worldwide [2]. Moreover, there are various conditions in which TIPS is not feasible or even recommendable [3]. TIPS is usually not used in the treatment of pre- and posthepatic PHT [4]. Specifically, TIPS is not suitable for patients with extrahepatic veno-occlusive disease, in which the vascular connection between splenic and portal circulation is interrupted, or for patients with advanced hepatic cirrhosis, because of the high risk of hepatic encephalopathy [5]. Contraindications to the placement of a TIPS include severe cardiac disease (e.g., congestive heart failure), multiple hepatic cysts, systemic infection/sepsis, or unrelieved biliary obstruction [6]. Nevertheless, these patients need to be treated, and PSSS is indicated to prevent variceal bleeding, spontaneous bacterial peritonitis, hepatorenal syndrome, and progressive liver failure [3, 7–14].

Due to the widespread use of TIPS, PSSS is becoming less common and experience with this operation is diminishing. There are many surgical portosystemic (PS) shunt options to consider in the preoperative planning (e.g., portacaval, mesocaval, splenorenal). The different PSSS techniques modify hepatopetal blood flow in different ways and can be categorized into nonselective, partially selective, and selective types. Nonselective PS shunt techniques establish complete drainage of portal as well as mesenterial blood flow into the inferior vena cava. Partially selective shunt techniques maintain the PS pressure gradient and thus ensure residual blood flow in the portal vein. Selective techniques separate the downgradient via esophageal collaterals from the portal hypertensive system and therefore have the smallest effects on portal perfusion. Interestingly, there are no long-term differences in terms of recurrent bleeding, hepatic encephalopathy, or overall survival between the different shunt techniques [15, 16]. This is in part attributable to a loss of selectivity over the years [17]. Since there is no “standard” shunt, the anatomy of the individual must be evaluated in each case to select the most appropriate shunting procedure. Careful planning, which shunting vessels and anastomosis (e.g., end-to-side, side-to-side) to choose, is necessary to avoid shunt failure or hepatic encephalopathy caused by (too) high shunt volume. Another shunt technique besides the more common shunt variants already mentioned is the mesoportal (Meso-Rex) shunt, which actually is a bypass rather than a true PS shunt. Meso-Rex shunts are of special interest in children and younger patients with extrahepatic portal occlusion. They maintain liver function and hepatopetal flow, therefore mostly avoiding hypersplenism, coagulopathies, and hyperammonemia while at the same time allowing normal neurological development to continue [18–21].

Nonetheless, PSSS is invasive and is associated with the intra- and perioperative risks of major abdominal vascular surgery—its major disadvantage in comparison to TIPS. Therefore, precise preoperative imaging and planning are of utmost importance.

The purpose of this study was to evaluate whether CT and MRI are sufficiently accurate and reliable for the preoperative planning of PSSS in patients who are not suitable for a TIPS procedure. With decreasing surgeon experience caused by the widespread of TIPS, a radiologist may be in an important position to assist the surgical team with pre-surgical planning to help guide patient management.

## Material and methods

### Patient population

**Fig. 1:**
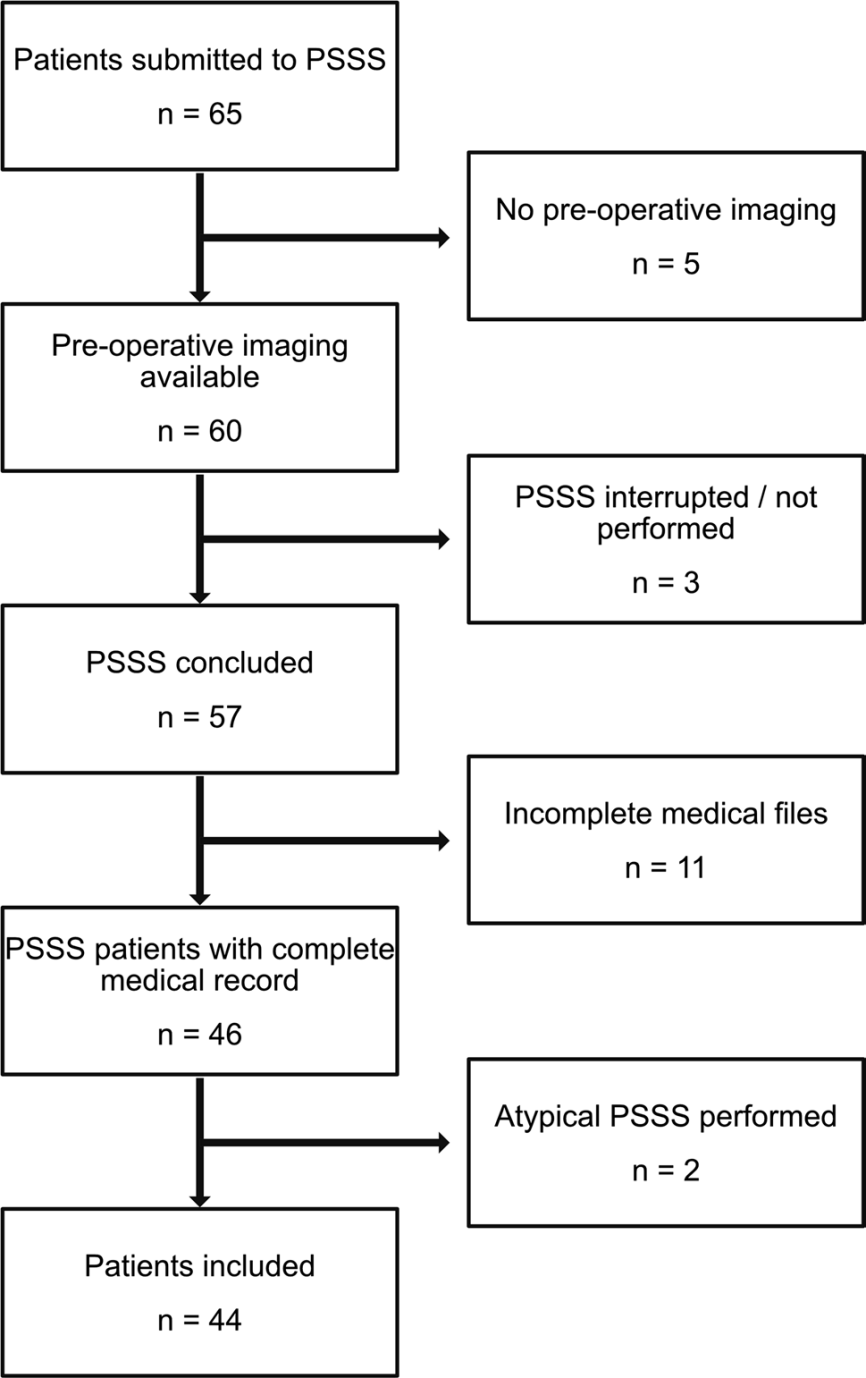
Flowchart of retrospective enrollment

Sixty-five patients who underwent PSSS between March 2002 and September 2013 were retrospectively identified from the surgical database of our hospital. Surgical reports, histology reports, cross-sectional imaging datasets, laboratory results, and medical records were obtained to generate the endpoint parameter. The patients underwent PSSS after having been classified as unsuitable for TIPS procedure in an interdisciplinary discussion. The main reason for not choosing TIPS was prehepatic PHT with chronic extrahepatic portal vein occlusion in 31 patients (70%). In seven pediatric patients with patent portal venous flow, PSSS was preferred due to the still incomplete body growth. In two patients, PSSS was indicated because of chronic TIPS occlusion. One patient was treated with PSSS during a hemicolectomy for cecum carcinoma to avoid a further intervention. Another patient was unsuitable for TIPS because of a previously performed right hepatic trisectionectomy because of a Klatskin’s tumor. In the other two patients, PSSS was preferred because of vascular anomalies (intrahepatic portal vein dysplasia and a mesenteric arteriovenous malformation). Nineteen of the initially identified patients were excluded because of incomplete medical records or lack of preoperative imaging. Two patients underwent an atypical shunt procedure, which was not part of the retrospective imaging-based evaluation. These two patients also had to be excluded. Forty-four patients were finally included (Fig. 1).

Our Institutional Review Board approved the study protocol including adult and pediatric patients, waiving informed consent (Application Number EA1/148/14) because of the retrospective study design.

### Imaging (CT and MRI)

Multiphasic contrast-enhanced CT [with iopromide (Ultravist 370®, Bayer Schering Pharma) or with iobitridol (Xenetic 350®-Guerbet GmbH)] was performed on a 16- or 64-slice CT scanner (Light Speed Power 16 or VCT 64; GE Medical Solutions, Fairfield, CT) using a triple-phase acquisition technique with arterial (~ 15-s delay), portal venous (~ 40-s delay), and equilibrium phase (~ 80-s delay) acquisition. The primary slice thickness was 0.625 mm. Tube voltage was 120 kV, tube current was modulated automatically based on a noise index of 15 and a maximally allowed current of 350 mA. Maximum intensity projections (MIPs) were reconstructed for each contrast phase.

Contrast-enhanced MRI was performed with gadoterate meglumine (Dotarem®-Guerbet GmbH) or gadobutrol (Gadovist®-Bayer Schering Pharma) at 1.5 T (Siemens Magnetom Avanto, Siemens Healthcare, Erlangen, Germany) using an eight-channel body phased-array surface coil. Due to the long observation period, the MRI protocols vary within the examined collective. All MRI protocols included multiphase dynamic contrast-enhanced T1-weighted sequences with fat saturation in axial (arterial, portal venous, venous, and delayed phase) and coronal orientation (venous phase).

### Planning of portosystemic shunt surgery

Preoperative images of all 44 patients—contrast-enhanced CT (*n* = 33) and/or MRI datasets (*n* = 15; *n* = 4 patients with both CT and MRI)—were analyzed on a dedicated PACS viewing workstation (Centricity, GE Healthcare, General Electric, Milwaukee, USA) by 2 radiologists with different levels of experience [Observer 1 (O1; T.D.): 12 years and Observer 2 (O2; J.S.): 4 years of experience in abdominal imaging]. Both observers were blinded to the surgical technique and outcomes.

The two observers evaluated patency and diameters of the portal venous system. Maximum diameters of the possible shunt vessels were measured at the suggested connection (down- and upstream) sites by the radiologists. The shortest distances between the possible shunt vessels [inferior vena cava (IVC), portal vein (PV), left and right portal vein branches, superior mesenteric vein (SMV), splenic vein (SV), and left renal vein (RV)] were measured.

Structures intervening between possible shunting vessels were identified in consensus by O1 and O2 as factors increasing surgical complexity (need for tissue resection before shunt creation). Other factors contributing to complexity included large vessel distance (> 20 mm) (necessity of graft interposition), thrombosis adjacent to connecting vessels (requires additional thrombectomy), and unavoidable use of a collateral vein instead of (occluded) major vein for establishing the shunt.

An algorithm for deciding about the surgical shunt technique based on clinical factors (e.g., age, indication, urgency of vascular decompression, vascular beds to relieve) has been proposed by the surgical team and is shown in Table 1. This algorithm takes the physiological properties of each shunt into account and was used in our retrospective analysis as a guideline rather than an absolute standard in assessing the recommendations made by our two observers.

**Table 1:** Institutional surgical algorithm that represents a synopsis of local surgeon preferences

Indication	Preferred shunt
Acute esophageal bleeding	Portacaval (end-to-side)
Extrahepatic portal thrombosis	Mesocaval, splenorenal (distal or side-to-side)
Extrahepatic portal thrombosis in children	Meso-Rex
Budd–Chiari syndrome	Portacaval or mesocaval
Ascites	Splenorenal (not distal)
Possible liver transplantation candidate	Portacaval or mesocaval

Based on the information available in this retrospective analysis, each of the two observers made a ranked recommendation (first and second choice) of two shunt techniques including the most appropriate anastomosis technique:

1. Splenorenal
  *Anastomosis* proximal end-to-side (Linton), distal end-to-side (Warren) or side-to-side (Cooley).
2. Portacaval
  *Anastomosis* end-to-side or side-to-side.
3. Mesocaval
  *Anastomosis* side-to-side.
4. Meso-Rex shunt.

Each technique proposed in our retrospective analysis was classified as “standard” or “complex”. If any factor of complexity was present, the procedure was classified as complex. During the decision process, the two observers were blinded to the later chosen surgical procedure. The imaging-based recommendations made by the two observers were compared with the shunt procedures actually accomplished by the surgeons treating the patients included in our analysis.

The success of PSSS was evaluated during postoperative hospitalization until discharge (< 30 days). PSSS failure was defined as early shunt occlusion or occurrence of major complications (rebleeding, organ failure, death).

To correlate the outcome of PSSS with vessel diameters, we calculated the shunt vessel ratio using the following formula:

$${\text{diameter}}_{{{\text{distal}}\;{\text{shunt}}\;{\text{vessel}}}} /{\text{diameter}}_{{{\text{proximal}}\;{\text{shunt}}\;{\text{vessel}}}}$$

The shunt vessel ratio, diameter of the smaller shunt vessel, and distance of the connected vessels were correlated with PSSS outcome.

### Statistical analysis

Statistical analysis was performed using SPSS Statistics Version 22 (IBM, Armonk, NY, USA). The *χ*^2^ test was used to assess differences in the accuracy of CT and MRI in preoperative planning. Cohen’s *κ* was used to evaluate inter-rater reliability. Continuous variables from two independent samples were evaluated using the Mann–Whitney *U* test. Receiver operating characteristic curve analysis was used to investigate binary classifiers. *p* values < 0.05 were considered statistically significant.

## Results

The collective consisted of 19 (43%) pediatric patients (0–21 years) with 3 infants (< 2 years), 8 children (2 to 12 years), and 8 adolescents (12 to 21 years) [22]. The mean age in adult patients (*n* = 25) was 44 years. Both genders were equally represented (22 each) in the study population. Table 2 summarizes diagnoses underlying PHT, signs and clinical findings on admission, and major indications for surgery. The average MELD score (applicable to patients ≥ 12 years) was 12 (range 7 to 24). For patients below 12 years of age, the average PELD score was 1.7 (range 0 to 10.9). In the overall collective, the average serum albumin level was 3.42 g/dl (range 2.0–5.1) [23].

**Table 2:** Characteristics of the study patients

	*n*	%
Major underlying causes of PHT		
Extrahepatic portal vein thrombosis	31	70
Associated with liver cirrhosis	7	
Other causes (e.g., clotting disorder)	24	
Liver cirrhosis	16	36
Metabolic/toxic	13	
Viral hepatitis	2	
Autoimmune hepatitis	1	
Wilson’s disease	1	2
Rendu–Osler–Weber disease	1	2
Budd–Chiari syndrome	1	2
Post-hemihepatectomy lymph fistula	1	2
Signs and clinical findings in PHT		
Esophagogastric varices	40	91
Splenomegaly	31	70
Ascites	19	43
Advanced stage symptoms^a^	3	7
Complications of PHT and indication for PSSS		
Varices with previous bleeding episode	25	57
Acute variceal bleeding	2	
Splenomegaly and secondary thrombocytopenia	19	43
Excessive ascites	5	11

*PHT* portal hypertension, *PSSS* portosystemic shunt surgery

^a^Hepatorenal syndrome, hepatic encephalopathy, spontaneous bacterial peritonitis

**Fig. 2:**
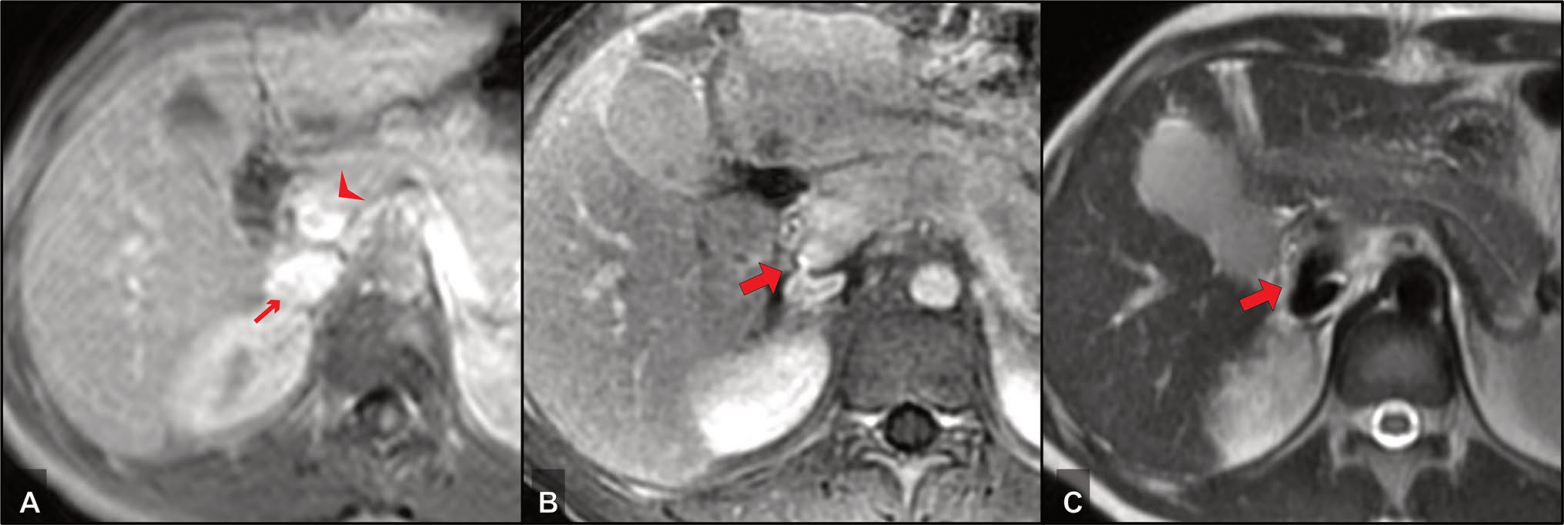
A 17-year-old female PHT patient with Wilson’s disease and recurrent variceal bleeding—PSSS procedure: portacaval side-to-side; **a** preoperative MRI, post-contrast T1-w, **b** postoperative MRI, post-contrast T1-w and **c** postoperative MRI, T2w. Small arrow: IVC; arrowhead: portal vein; bold arrow: portacaval anastomosis

**Fig. 3:**
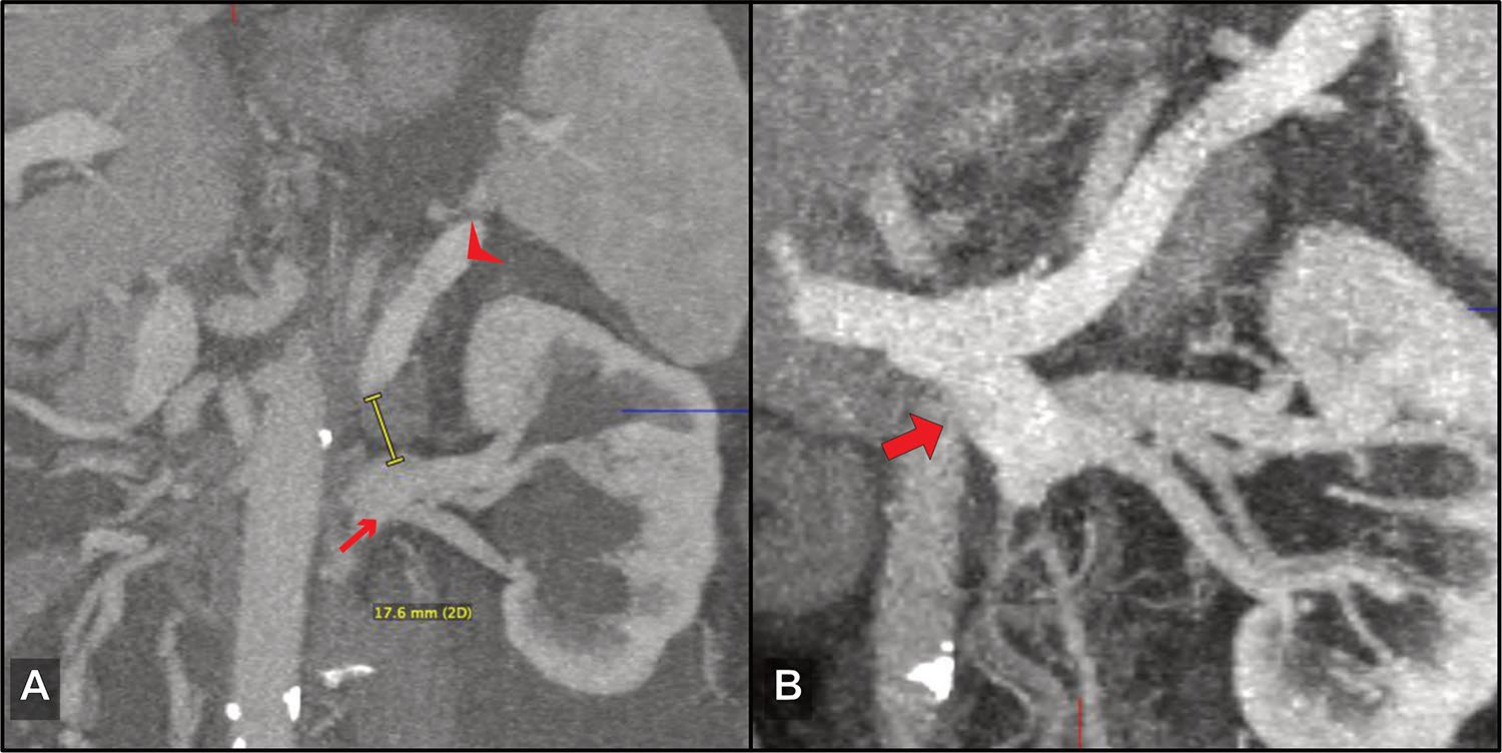
A 71-year-old male PHT patient with excessive ascites after extended right hemihepatectomy (diagnosis: intrahepatic cholangiocarcinoma)—PSSS: splenorenal side-to-side; **a** preoperative CT, oblique MIP reconstruction and **b** postoperative CT, oblique MIP reconstruction. Small arrow: left renal vein; arrowhead: splenic vein; bold arrow: splenorenal anastomosis

**Fig. 4:**
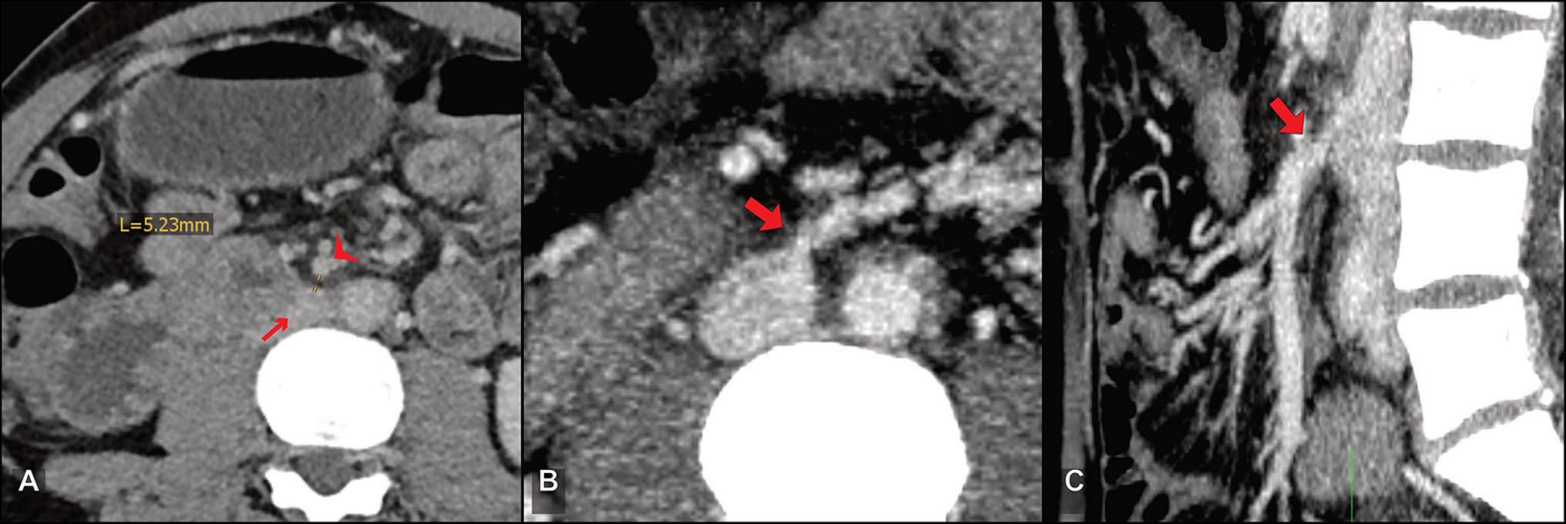
A 49-year-old male PHT patient with liver cirrhosis, extrahepatic portal vein thrombosis, and advanced symptoms—PSSS procedure: mesocaval; **a** preoperative CT, **b** postoperative CT, axial MIP reconstruction and **c** postoperative CT, sagittal MIP reconstruction. Small arrow: IVC; arrowhead: SMV; bold arrow: mesocaval anastomosis

Eighteen patients had portacaval PSSS (Fig. 2), in which end-to-side was the most commonly used anastomosis. Splenorenal (Fig. 3) and mesocaval (Fig. 4) PSSS were performed in 15 and 11 cases, respectively (Table 3). A Meso-Rex shunt was not chosen by the surgeons in our evaluated cases. However, as it was part of the available options to the radiologists reading, it is still mentioned in Table 3. All PSSS procedures included in this study were performed or at least supervised by the same surgeon in charge.

**Table 3:** Types of portosystemic shunts performed in the study population

Shunt	*n*	%
Splenorenal	15	34
Side-to-side (Cooley)	10	
Distal (Warren)	3	
Proximal (Linton)	2	
Portacaval	18	41
End-to-side	12	
Side-to-side	6	
Mesocaval	11	25
Side-to-side	11	
Meso-Rex	0	0

In terms of the vessels (splenorenal, portacaval, mesocaval, and Meso-Rex) used for PSSS, accuracy of the two observers was 73%/80% (CT/MRI) for O1 and 52%/60% for O2 if only their first choices were taken into consideration. Inter-rater reliability for the first choices was fair (Cohen’s *κ* = 0.271, *p* = 0.006). If the shunt technique actually used matched the first or second choice, accuracy increased to 88%/100% (O1) and 76%/73% (O2). Evaluation of recommended shunt vessels and the way they should be anastomosed (e.g., end-to-side, side-to-side, proximal, distal) showed an accuracy of 64%/53% (O1) and 36%/33% (O2) if only the first choice was considered. Inter-rater reliability for the first choices of vessels and anastomosis was fair (Cohen’s *κ* = 0.257, *p* < 0.001). If the shunt vessel and anastomosis technique matched the proposed first two choices, accuracy increased to 79%/72% (O1) and 67%/60% (O2). There was no significant difference in accuracy between MRI- and CT-based recommendations for either observer (*p* > 0.05, Table 4). PSSS procedures and radiological recommendations for each patient are displayed in Supplementary Data.

**Table 4:** Accuracy in predicting shunt procedure including anastomotic variants

	O1	O2
Accuracy of shunt procedures recommended on the basis of CT and/or MRI		
1st option		
CT	73%	52%
MRI	80%	60%
*p* value	0.728	0.756
1st + 2nd option		
CT	88%	76%
MRI	100%	73%
*p* value	0.294	1.000
Accuracy of suggested shunts and proposed anastomotic variant		
1st option		
CT	64%	36%
MRI	53%	33%
*p* value	0.538	1.000
1st + 2nd option		
CT	79%	67%
MRI	73%	60%
*p* value	0.720	0.749

Surgeons actually chose a vessel connection which was not included among the first 2 choices by O1 in 5 cases (11%) and by O2 in 12 cases (26%).

**Fig. 5:**
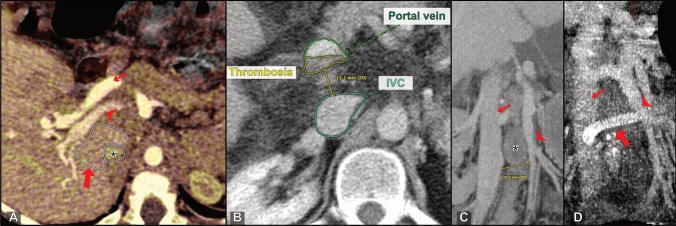
Factors of complexity. **a** Oblique axial CT (fused portal venous phase and venous phase) shows an intervening caudate lobe (PSSS procedure: portacaval end-to-side with subsegmental liver resection); bold arrow: caudate lobe; arrowhead: portal vein; small arrow: hepatic artery; asterisk: IVC. **b** Axial CT with partial thrombosis of the extrahepatic portal vein (PSSS procedure: portacaval side-to-side after thrombectomy). **c** Oblique coronal CT MIP shows a large distance of 29 mm between IVC (small arrow) and superior mesenteric vein (arrowhead). In this patient, an allograft was interposed as seen in **d** (PSSS procedure: mesocaval with graft interposition). **d** Postoperative oblique coronal CT MIP reconstruction shows the interposed graft (bold arrow) connecting the SMV (arrowhead) and IVC (small arrow)

In 15 patients, surgeons chose a PSSS technique that was classified as complex by the radiologist. In 13 (87%) of these patients, complexity of the procedure was confirmed by the operative report. The retrospective imaging-based evaluation had 80% sensitivity and 96% specificity in predicting complex surgical procedures identified in consensus by the two radiologists. In two patients, resection of interposing structures was deemed necessary by the radiologists. In one of these cases, segmental liver resection (segment 1 according to the Couinaud classification) was performed (Fig. 5a). In the second case, embolization of an intervening arteriovenous malformation was performed in preparation for PSSS. In a third patient, a segmental liver resection (segment 1) that was not suggested by the radiology observers was performed. Partial thrombosis of the connecting vessels was identified by the radiologists in 4 patients, and thrombectomy was performed in 3 (75%) of these patients (Fig. 5b). Use of a collateral vessel was proposed and also performed in one patient (100%). In 9 patients, the distance between the connecting vessels was > 20 mm, and graft interposition was recommended by the observers. 8 (89%) of these patients actually received a graft (Fig. 5c, d).

**Fig. 6:**
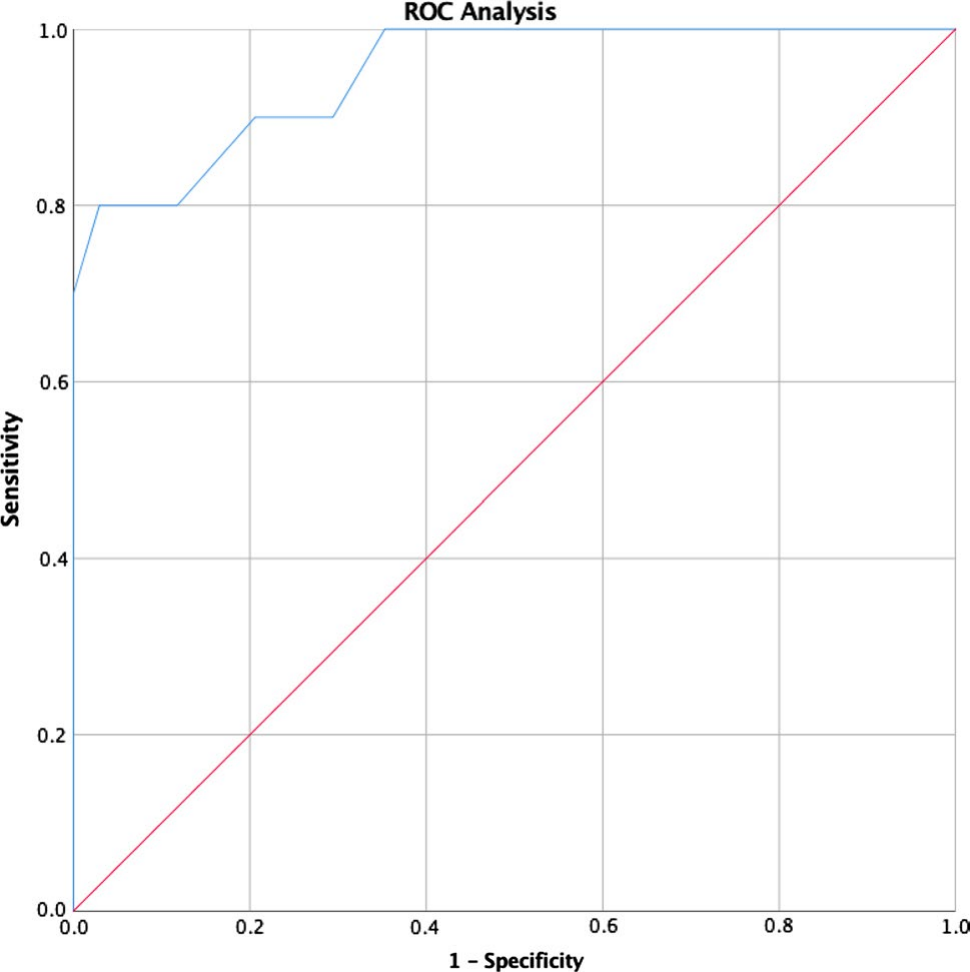
ROC analysis—distance of connected vessels and need for graft interposition; AUC 0.950 (*p* < 0.001); max. Youden index 0.771 at 20 mm

Overall, graft interposition (splenorenal *n* = 3; portacaval *n* = 2; mesocaval *n* = 5) was performed in 10 patients of our study population. ROC analysis of vessel distances and graft use showed an AUC of 0.950 (*p* < 0.001, Fig. 6). According to the Youden index, the optimal cutoff was 20 mm (Youden index 0.771). Only one patient with a distance of > 20 mm did not receive a graft interposition, and this patient developed early shunt occlusion.

PSSS was successful in 38 patients (82%), and their shunts were perfused until discharge. Eight patients (18%) showed therapy failure/early shunt occlusion (< 30 days after shunt surgery). Three of these patients (shunt indication: acute variceal bleeding) died during hospitalization.

Analysis of PSSS outcome revealed a significant correlation between shunt vessel distance and early occlusion. The larger the distance, the higher the risk of early shunt occlusion (*p* = 0.030). There was no significant correlation between successful PSSS and the diameter of smaller connecting vessels or the shunt vessel ratio (*p* > 0.05, Table 5).

**Table 5:** Analysis of correlation of shunt vessel diameter and distance with early success and failure/shunt occlusion (< 30 days)

Outcome of PSSS during hospitalization (<30 days)							
	Success (*n* = 36)			Failure (*n* = 8)			Significance
	Mean	SD	Range	Mean	SD	Range	
Distance between vessels (mm)	12.67	11.69	56.00	20.38	10.87	33.00	*p* = 0.030
Small shunt vessel diameter (mm)	10.17	4.39	21.00	10.25	3.01	9.00	*p* = 0.709
Shunt vessel ratio	0.734	0.567	2.742	0.500	0.156	0.475	*p* = 0.482

## Discussion

Despite the success story of TIPS [2], PSSS can have benefits in the treatment of advanced PHT [5]. We retrospectively analyzed 44 patients who underwent PSSS at our center because TIPS was no adequate treatment option. Longer-term results available for the different shunting techniques suggest that preoperative diagnostic workup should focus on vascular anatomy [15]. This is the rationale for the study presented here, which, to the best of our knowledge, is the first study focusing on the role of preoperative imaging for predicting the most suitable approach of PSSS. Our analysis shows that contrast-enhanced CT and MRI are suitable for the imaging-based planning of surgical shunt creation and at the same time allow identification of complicating circumstances. The results also show that interpretation of the complex imaging findings and the consecutive planning of the surgical approach require an experienced radiologist and may also require a multidisciplinary discussion to choose the most appropriate surgical management.

With its higher periprocedural morbidity due to advanced liver disease, PSSS requires adequate preoperative planning to avoid extensive surgical exploration [24]. With the high-resolution cross-sectional imaging modalities now available, adequate planning of the most suitable shunt should be possible even in patients with advanced PHT. Adequate evaluation of portal venous anatomy for reliable imaging-based surgical planning has been shown in patients undergoing liver transplantation and pancreatic surgery [25, 26]. While it has been shown that precise puncture guidance in TIPS procedures is possible with contrast-enhanced CT and the option of 3D reconstruction [27, 28], similar data for the imaging-based planning of PSSS are not available, and our study was conducted to fill this gap. Besides choosing between TIPS and PSSS, the combination of both is a feasible alternative for complex cases with extrahepatic disease and should be kept in mind in the pre-interventional planning [29]. In our collective, the main reason to prefer PSSS to TIPS in adults was a chronic extrahepatic portal vein occlusion. However, TIPS as a possibility should not be excluded per se in these patients, since TIPS can also help in the treatment of extrahepatic portal vein occlusion [30].

Our results show that an experienced reader can (retrospectively) predict which surgical technique was going to be used by an experienced surgeon with high accuracy in both CT (88%) and MRI (100%). A less-experienced reader achieves lower accuracies of about 75% with both modalities, which is still adequate, though, the fair inter-rater reliability of their first choices underlines the need for an experienced radiologist in the interpretation of the complex preoperative imaging. Moreover, our findings suggest that there are no significant differences in the preoperative accuracy of MRI and CT. These findings support an earlier study reporting the good performance of CT venography in the evaluation of portosystemic collateral vessels [31]. In only 10% of the patients included in our retrospective analysis did the surgeon choose a vessel connection not recommended by the experienced reader. The high incidence of therapy failure (40%) in these cases indicates that deviation from preoperative imaging findings could lead to higher rates of shunt failure and consecutively increased morbidity. Similarly, it has been shown that preoperative CT assessment improves intraoperative management of portosystemic shunts during liver transplantation [32].

In addition to providing information on vascular anatomy, MRI and CT identify complicating factors that require additional surgical techniques. Caudate lobe enlargement is common in cirrhosis of different etiologies and is frequently found in patients with PHT requiring PSSS [33]. Therefore, a hypertrophic caudate lobe has to be considered as a complicating factor in the planning of potential portacaval shunt surgery. Our study shows that imaging reliably identifies interfering structures, which can thus be considered in preoperative planning. Large distances between connecting vessels sometimes require the use of interposition grafts. General recommendations regarding the vein distance above which graft interposition should be performed do not exist. Our analysis identified a vessel distance of 20 mm as the optimal cutoff for use of an interposition graft by experienced surgeons. In addition to its use as a criterion for deciding about the need for an interposition graft, vessel distance was identified as the only risk factor for early shunt failure/occlusion in our cohort. In contrast, the diameters of the veins connected for shunt creation did not correlate with early shunt occlusion. The observed incidence of surgical failure/shunt occlusion (17%) in our patients is attributable to the wide range of shunt indications and a high percentage of advanced and acute cases with poor hepatic reserve compared to other studies [34, 35].

Our study has some limitations. Despite the retrospective nature and the small sample size, only patients who underwent surgery were included in our analysis. This is attributable to the fact that PSSS is increasingly being replaced by TIPS. The surgeons performing PSSS were also aware of the preoperative imaging findings; however, there was no documentation how the imaging findings influenced the surgeons’ choice of shunt procedure. Practice patterns and surgeons’ experience are likely variable between institutions, so that the results of the study may not be applicable to other sides. There are various confound factors that could influence the prediction. The surgeons’ personal preferences may have played a role in the choice of PSSS variants, and these may have changed over the 11-year study period. The more experienced radiologist could have been better at the prediction because he knew the surgeons and their preferred techniques more intimately. To minimize these effects, we evaluated the first two radiological choices instead of only using a single recommendation. Our results could pave the way for a prospective study, which is needed, to answer the question if pre-surgical imaging provides adequate and important information and impacts patient outcomes.

In conclusion, preoperative cross-sectional imaging and an experienced radiologist can guide the surgeon in PSSS. Imaging is not only inevitable to provide accurate planning in general but rather to reliably exclude unfavorable shunt variants. Optimized CT and MRI examinations provide the information necessary to identify technically feasible alternatives and complicating factors (which may require an interposition graft, resection of intervening structures, or prior thrombectomy).

## Electronic supplementary material

Below is the link to the electronic supplementary material.

Supplementary material 1 (DOCX 24 kb)

## Abbreviations

IVC: Inferior vena cava
O1: Observer one (more experienced)
O2: Observer two (less experienced)
PHT: Portal hypertension
PSSS: Portosystemic shunt surgery
PV: Portal vein
SMV: Superior mesenteric vein
TIPS: Transjugular intrahepatic portosystemic shunt
