# Correction to: Portosystemic shunt surgery in the era of TIPS: imaging‑based planning of the surgical approach

**DOI:** 10.1007/s00261-021-03153-1

**Published:** 2021-06-22

**Authors:** Uli Fehrenbach, Safak Gul‑Klein, Miguel de Sousa Mendes, Ingo Steffen, Julienne Stern, Dominik Geisel, Gero Puhl, Timm Denecke

**Affiliations:** 1 Department of Radiology, Charité – Universitätsmedizin Berlin, Augustenburger Platz 1, 13353 Berlin, Germany; 2 Department of Surgery, Campus Charité Mitte | Campus Virchow-Klinikum, Charité – Universitätsmedizin Berlin, corporate member of Freie Universität Berlin, Humboldt-Universität Zu Berlin, and Berlin Institute of Health, Berlin, Germany; 3 Department of Radiology, Krankenhaus Nordwest, Frankfurt am Main, Germany; 4 Department of Surgery, Asklepios Klnik Altona, Hamburg, Germany; 5 Department of Diagnostic and Interventional Radiology, Universitätsklinikum Leipzig, Leipzig, Germany

## Correction to: Abdominal Radiology (2020) 45:2726–2735 10.1007/s00261-020-02599-z

The article “Portosystemic shunt surgery in the era of TIPS: imaging-based planning of the surgical approach”, written by Uli Fehrenbach, Safak Gül-Klein, Miguel de Sousa Mendes, Ingo Stefen, Julienne Stern, Dominik Geisel, Gero Puhl, Timm Denecke, was originally published electronically on the publisher’s internet portal on 5 June 2020 without open access. With the author(s)’ decision to opt for Open Choice the copyright of the article changed on 24 May 2021 to © The Author(s) 2020 and the article is forthwith distributed under a Creative Commons Attribution 4.0 International License, which permits use, sharing, adaptation, distribution and reproduction in any medium or format, as long as you give appropriate credit to the original author(s) and the source, provide a link to the Creative Commons licence, and indicate if changes were made. The images or other third party material in this article are included in the article’s Creative Commons licence, unless indicated otherwise in a credit line to the material. If material is not included in the article’s Creative Commons licence and your intended use is not permitted by statutory regulation or exceeds the permitted use, you will need to obtain permission directly from the copyright holder. To view a copy of this licence, visit http://creativecommons.org/licenses/by/4.0.

The original article has been corrected.

