# Supplementary material for: Portosystemic shunt surgery in the era of TIPS: imaging-based planning of the surgical approach
Source: Abdom Radiol (NY). 2020 Jun 5;45(9):2726–35. doi: 10.1007/s00261-020-02599-z (PMC8197708; doi:10.1007/s00261-020-02599-z)
Supplement: Supplementary file 1 — Supplementary material 1 (DOCX 24 kb) [file 261_2020_2599_MOESM1_ESM.docx]

|  | Surgery | | | Radiology | | | | | |
| --- | --- | --- | --- | --- | --- | --- | --- | --- | --- |
| ID | PSSS (anastomosis) | Complex | Factor of Complexity | Modality | Observer 1 | | Observer 2 | | Complex |
|  |  |  |  |  | First | Second | First | Second |  |
| 1 | portocaval (E/S) | No |  | CT | portocaval (E/S) | mesocaval (S/S) | splenorenal (S/S) | splenorenal (distal) | No |
| 2 | Atypical | No |  | MRI | splenorenal (S/S) | splenorenal (proximal) | splenorenal (distal) | splenorenal (S/S) | No |
| 3 | mesocaval (S/S) | Yes | Graft | MRI | mesocaval (S/S) | splenorenal (S/S) | Meso-Rex | mesocaval (S/S) Int | No |
| 4 | portocaval (S/S) | No |  | CT | portocaval (S/S) | splenorenal (S/S) | Meso Rex | portocaval (S/S) | No |
| 5 | portocaval (E/S) | Yes | Resection & Thrombectomy | CT | portocaval (E/S) | splenorenal (S/S) | splenorenal (distal) | splenorenal (S/S) | Yes |
| 6 | splenorenal (proximal) | Yes | Thrombectomy | CT & MRI | splenorenal (S/S) | mesocaval (S/S) | splenorenal (S/S) | splenorenal (distal) | Yes |
| 7 | mesocaval (S/S) | No |  | MRI | mesocaval (S/S) | splenorenal (S/S) | mesocaval (S/S) | portocaval (S/S) | No |
| 8 | splenorenal (S/S) | Yes | Graft | CT & MRI | splenorenal (S/S) | mesocaval (S/S) | splenorenal (distal) | splenorenal (S/S) | Yes |
| 9 | mesocaval (S/S) | No |  | CT | mesocaval (S/S) | splenorenal (S/S) | mesocaval (S/S) | splenorenal (distal) | No |
| 10 | mesocaval (S/S) | No |  | CT | mesocaval (S/S) | splenorenal (S/S) | mesocaval (S/S) | portocaval (S/S) | No |
| 11 | portocaval (S/S) | Yes | Graft | CT | mesocaval (S/S) | splenorenal (S/S) | mesocaval (S/S) | portocaval (S/S) | Yes |
| 12 | mesocaval (S/S) | Yes | Graft | CT | mesocaval (S/S) | splenorenal (S/S) | splenorenal (distal) | splenorenal (S/S) | Yes |
| 13 | splenorenal (distal) | No |  | CT | splenorenal (distal) | mesocaval (S/S) | mesocaval (S/S) | splenorenal (distal) | No |
| 14 | mesocaval (S/S) | Yes | Graft | CT | portocaval (S/S) | Splenorenal (S/S) | splenorenal (S/S) | splenorenal (distal) | Yes |
| 15 | splenorenal (S/S) | No |  | MRI | splenorenal (S/S) | splenorenal (distal) | splenorenal (S/S) | splenorenal (distal) | Yes |
| 16 | splenorenal (S/S) | No |  | CT & MRI | splenorenal (S/S) | splenorenal (distal) | splenorenal (S/S) | splenorenal (distal) | No |
| 17 | portocaval (E/S) | No |  | MRI | portocaval (S/S) | splenorenal (distal) | splenorenal (S/S) | splenorenal (distal) | No |
| 18 | mesocaval (S/S) | Yes | Graft | MRI | mesocaval (S/S) | splenorenal (S/S) | SR atypisch | splenorenal (proximal) | Yes |
| 19 | mesocaval (S/S) | No |  | CT | mesocaval (S/S) | splenorenal (proximal) | splenorenal (distal) | mesocaval (S/S) | No |
| 20 | mesocaval (S/S) | No |  | CT | splenorenal (S/S) | mesocaval (S/S) | mesocaval (S/S) | splenorenal (S/S) | Yes |
| 21 | Atypical | No |  | CT | mesocaval (S/S) | splenorenal (S/S) | mesocaval (S/S) | splenorenal (S/S) | No |
| 22 | portocaval (E/S) | No |  | CT | splenorenal (proximal) | mesocaval (S/S) | portocaval (E/S) | mesocaval (S/S) | No |
| 23 | splenorenal (distal) | No |  | CT | splenorenal (proximal) | splenorenal (distal) | splenorenal (distal) | mesocaval (S/S) | No |
| 24 | portocaval (E/S) | Yes | Resection | CT | portocaval (E/S) | splenorenal (S/S) | splenorenal (distal) | portocaval (S/S) | No |
| 25 | splenorenal (S/S) | No |  | CT | splenorenal (S/S) | mesocaval (S/S) | portocaval (E/S) | mesocaval (S/S) | No |
| 26 | portocaval (E/S) | No |  | CT | portocaval (E/S) | splenorenal (proximal) | portocaval (E/S) | portocaval (S/S) | No |
| 27 | splenorenal (S/S) | No |  | MRI | splenorenal (distal) | mesocaval (S/S) | splenorenal (distal) | splenorenal (S/S) | No |
| 28 | splenorenal (proximal) | No |  | MRI | mesocaval (S/S) | splenorenal (proximal) | mesocaval (S/S) | splenorenal (proximal) | No |
| 29 | portocaval (S/S) | No |  | CT | splenorenal (S/S) | splenorenal (proximal) | mesocaval (S/S) | portocaval (S/S) | No |
| 30 | splenorenal (S/S) | No |  | CT | splenorenal (distal) | splenorenal (S/S) | splenorenal (distal) | splenorenal (S/S) | No |
| 31 | portocaval (E/S) | Yes | Resection (AVM) | CT | portocaval (E/S) | splenorenal (S/S) | portocaval (E/S) | splenorenal (S/S) | Yes |
| 32 | mesocaval (S/S) | Yes | Graft | CT | splenorenal (proximal) | splenorenal (S/S) | mesocaval (S/S) | splenorenal (S/S) | Yes |
| 33 | splenorenal (S/S) | Yes | Graft | MRI | splenorenal (S/S) | splenorenal (proximal) | splenorenal (S/S) | splenorenal (distal) | Yes |
| 34 | splenorenal (S/S) | No |  | MRI | splenorenal (S/S) | mesocaval (S/S) | splenorenal (S/S) | splenorenal (distal) | No |
| 35 | splenorenal (S/S) | Yes | Graft | CT | portocaval (S/S) | splenorenal (S/S) | splenorenal (distal) | splenorenal (S/S) | No |
| 36 | portocaval (E/S) | Yes | Thrombectomy | CT | portocaval (E/S) | mesocaval (S/S) | portocaval (S/S) | mesocaval (S/S) | Yes |
| 37 | portocaval (E/S) | No |  | MRI | portocaval (E/S) | splenorenal (distal) | splenorenal (S/S) | splenorenal (distal) | No |
| 38 | portocaval (S/S) | Yes | Collateral Vessel | CT | portocaval (S/S) | splenorenal (S/S) | splenorenal (distal) | portocaval (S/S) | Yes |
| 39 | splenorenal (distal) | No |  | CT | portocaval (E/S) | splenorenal (S/S) | portocaval (E/S) | splenorenal (S/S) | No |
| 40 | portocaval (S/S) | No |  | CT | portocaval (S/S) | splenorenal (S/S) | portocaval (S/S) | mesocaval (S/S) | No |
| 41 | mesocaval (S/S) | No |  | CT | mesocaval (S/S) | splenorenal (proximal) | splenorenal (distal) | splenorenal (S/S) | No |
| 42 | portocaval (E/S) | No |  | CT | splenorenal (S/S) | portocaval (E/S) | splenorenal (distal) | splenorenal (proximal) | No |
| 43 | portocaval (E/S) | No |  | CT | portocaval (E/S) | mesocaval (S/S) | portocaval (E/S) | mesocaval (S/S) | No |
| 44 | portocaval (S/S) | Yes | Graft | CT | portocaval (S/S) | splenorenal (S/S) | splenorenal (S/S) | splenorenal (distal) | Yes |
| 45 | portocaval (E/S) | No |  | MRI | splenorenal (S/S) | portocaval (E/S) | splenorenal (S/S) | splenorenal (distal) | No |
| 46 | splenorenal (S/S) | No |  | CT & MRI | splenorenal (S/S) | mesocaval (S/S) | splenorenal (proximal) | splenorenal (S/S) | No |

Supplementary Data: Performed PSSS procedures and radiological recommendations for each patient

PSSS: Portosystemic Shunt Surgery; S/S: Side-to-side; E/S: End-to-side
